# Microstructure and Shear Strength of SiC Joints Brazed with a Si–Ti–Al Filler Alloy

**DOI:** 10.3390/ma19183990

**Published:** 2026-09-19

**Authors:** Lianfeng Wei, Zhuyue Lv, Rui Xu, Yumin Zhao, Ce Wang, Yong Zheng, Xuehan Li

**Affiliations:** 1 National Key Laboratory for Science and Technology on Reactor and Materials, Nuclear Power Institute of China, Chengdu 610041, China; wfenghit@163.com (L.W.); npic_zhengy@126.com (Y.Z.); npic_lixh@163.com (X.L.); 2 Zhengzhou Advanced Research Institute, Harbin Institute of Technology, Zhengzhou 450000, China; lzy010611@163.com (Z.L.); xu060058@163.com (R.X.); 15994050903@163.com (Y.Z.)

**Keywords:** SiC, Si–Ti–Al filler, vacuum brazing, interfacial bonding mechanism, high-temperature strength

## Abstract

The joining of solid-state sintered silicon carbide (SiC) was achieved using a novel Si-Ti-Al ternary alloy via vacuum brazing. This study investigates a predominantly non-carbide-dominated interfacial bonding mechanism and the influence of brazing temperature on joint microstructure, mechanical properties, and high temperature reliability. The high Si content promoted the incorporation of Ti into Ti–Si phases within the brazed seam, thereby limiting the amount of Ti available for reaction with SiC. No continuous TiC layer was detected within the spatial resolution of the employed characterization methods. Minor discrete Al_4_C_3_ precipitates were identified at the interface but did not constitute the dominant bonding phase. Brazing at 1360 °C yielded an optimal microstructure featuring highly regular coral-like eutectic clusters, resulting in a peak room temperature shear strength of 102.8 MPa. The joints also exhibited favorable high temperature reliability, maintaining a shear strength of 58.4 MPa at 1000 °C. Microstructural analysis following thermal exposure revealed partial coarsening of primary blocky phases and interfacial Al_4_C_3_ precipitates via Ostwald ripening, which contributed to the reduction in high temperature strength. Nevertheless, the robust retention of fine eutectic clusters ensured satisfactory structural stability under thermal loading. This work provides a viable strategy for designing Si-based brazing fillers for high-performance SiC ceramic joining.

## 1. Introduction

With excellent mechanical and chemical properties at high temperatures, silicon carbide (SiC) has great potential applications in extreme environments such as aerospace, nuclear technology, and heat exchangers [1,2,3,4]. It is regarded as an ideal candidate for accident-tolerant fuel (ATF) cladding materials [5]. SiC-based cladding exhibits exceptionally outstanding high temperature steam oxidation resistance under beyond-design-basis accident conditions [6,7], along with a low neutron absorption cross-section and superior irradiation stability [8,9]. However, due to its high brittleness and challenging machinability, fabricating complex thin-walled tubular structures in a single sintering step is extremely difficult. Therefore, reliable joining technologies for macroscopic assembly have emerged as a highly necessary technical path for its engineering application [10].

Among the available joining methods, brazing is widely used for ceramic joining because of its process flexibility, adaptability to complex structures, and relatively low joining stress [11,12]. Nevertheless, controlling the interfacial reaction during high-temperature brazing remains challenging. Owing to the chemical inertness of the SiC surface, active elements such as Ti and Zr are commonly added to brazing fillers to improve wettability and promote interfacial bonding [13]. These active elements can react with SiC to form carbide- and silicide-containing interfacial reaction layers. For example, Liu et al. identified a continuous TiC layer and a discontinuous Ti_5_Si_3_ layer in SiC joints brazed with an Ag–Cu–In–Ti filler [13]. Their results showed that the joint strength initially increased and subsequently decreased with increasing reaction-layer thickness because excessive layer growth intensified the thermal-expansion mismatch and residual stress. Therefore, controlling the extent of the Ti–SiC interfacial reaction is important for achieving a balance between interfacial bonding and joint reliability. Accordingly, a Si-rich Si–Ti–Al filler system was designed in the present study to favor the incorporation of Ti into Ti–Si phases within the filler, with the aim of limiting excessive Ti–SiC interfacial reactions. However, this highly brittle carbide layer often acts as the source of extreme stress concentration during the cooling process, severely deteriorating the mechanical integrity of the joints. Furthermore, excessive interfacial reactions can lead to the severe decomposition of the SiC base material and the precipitation of harmful graphite phases that destroy interfacial strength [14]. Recently, non-carbide-dominated interfacial bonding mechanisms have attracted widespread attention in the academic community [15]. For instance, recent cutting-edge research has demonstrated that when a Si-rich Si-Pr eutectic filler is used to braze Reaction-Bonded Silicon Carbide, (RBSiC) no new phases are formed at the interface [16,17]. Studies have confirmed that the enriched Si in the filler effectively inhibits the side reactions between active elements and SiC, thereby preventing the formation of graphite at the interface [18,19]. Instead of relying on intermediate compound formation, this advanced bonding mechanism involves atomic bond formation facilitated by the diffusion of the filler and interface reconstruction, successfully achieving a high shear strength of 62.6 MPa. This novel metallurgical mechanism, which replaces “violent interfacial reactions” with “thermodynamic inhibition,” provides a vital inspiration for solving the high-temperature embrittlement issue in ceramic joints.

Inspired by these findings, this study investigates a Si–Ti–Al ternary filler system to clarify how filler composition and brazing temperature affect the interfacial microstructure and mechanical properties of SiC joints. The working hypothesis is that increasing the Si content of the filler may limit the formation of a continuous TiC reaction layer and graphite while maintaining effective interfacial bonding. This hypothesis is based on the possibility that the Si-rich filler promotes the formation of Ti-Si phases within the brazed seam, thereby reducing the amount of Ti available for reaction with SiC. Consequently, excessive interfacial reactions and SiC decomposition are expected to be reduced. The formation and distribution of Ti-containing phases in the filler and brazed joints were therefore examined to determine whether Ti was mainly incorporated into Ti-Si phases rather than forming a continuous TiC layer at the SiC interface. If Ti is primarily incorporated into Ti-Si phases within the filler, its reaction with SiC may be limited, thereby reducing the tendency to form a continuous TiC layer and graphite at the interface [20]. Meanwhile, the addition of Al lowers the melting temperature of the filler and influences the formation and distribution of the phases within the brazed seam. To examine the proposed hypothesis, four filler compositions were first characterized and compared in terms of their melting behavior, wettability, and microstructures using Differential Scanning Calorimetry (DSC), wetting tests, and Scanning Electron Microscope- Energy Dispersive X-ray Spectroscopy (SEM–EDS), while X-ray Diffraction (XRD) was used to identify the phase constituents of the representative filler. Based on the favorable balance among these characteristics, the 68Si filler was subsequently selected for SiC joining experiments, and the effects of brazing temperature on the joint microstructure and shear strength were evaluated.

The intended advantage of this composition design is to obtain an appropriate balance among melting behavior, wettability, interfacial integrity, and joint strength without relying on the formation of a continuous TiC-dominated reaction layer. This study establishes relationships among filler composition, brazing temperature, interfacial microstructure, and joint strength, providing guidance for the development of Si–Ti–Al fillers for SiC joining.

## 2. Materials and Methods

### 2.1. Materials Preparation

Pressureless-sintered SiC blocks supplied by Hefei Danjing Materials Technology Co., Ltd. (Hefei, China) were used as the substrates. The SiC blocks were machined into two specimen sizes: 5 × 5 × 3 mm^3^ and 8 × 8 × 3 mm^3^. Before brazing, the joining surfaces were ground using a 3000-grit diamond grinding disc (DiaRe, Trojan, Suzhou, Jiangsu, China) and subsequently polished using a 1 μm diamond polishing suspension. The surface topography of the polished SiC joining surfaces was characterized using a Dimension FastScan atomic force microscope (Bruker, Santa Barbara, CA, USA) over a scanning area of 50 × 50 μm^2^. The measured areal arithmetic mean height (Sa) and root-mean-square height (Sq) were 25.2 and 29.5 nm, respectively. The 5 × 5 × 3 mm^3^ SiC block was placed on the 8 × 8 × 3 mm^3^ SiC block, with the filler positioned between their joining surfaces to form a sandwich assembly. A uniaxial pressure of 0.1 MPa was applied normal to the joining surfaces during brazing.

**Figure 1:**
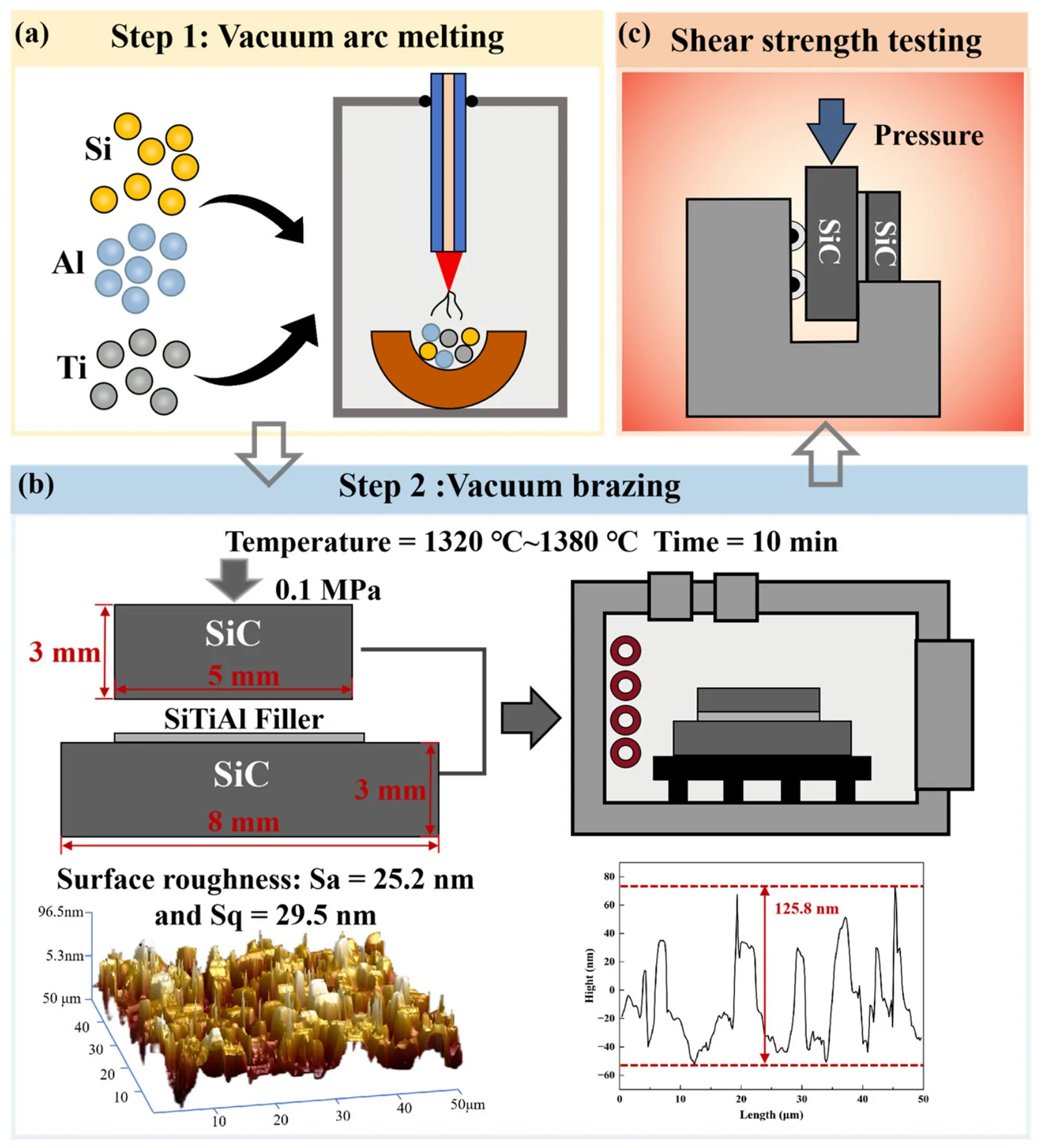
Schematic diagram of alloy melting, brazing assembly and joint shear test. (**a**) Melting diagram, (**b**) brazing assembly diagram, (**c**) shear test diagram.

Four Si–Ti–Al filler alloys with a constant Ti content of 20 wt.% were designed and designated as 72Si, 68Si, 64Si, and 60Si. Their nominal compositions are listed in Table 1. To ensure chemical homogeneity, the filler metals were prepared by arc melting pure Si, Ti, and Al blocks in a water-cooled copper hearth under a high-purity argon atmosphere (Figure 1a). The alloy ingots were remelted multiple times and subsequently cut into thin foils for brazing. For each brazed joint, a single filler foil with a nominal area of approximately 5 × 5 mm^2^ and a thickness of approximately 0.1 mm was placed between the joining surfaces of the two SiC blocks. The same nominal foil area, thickness, and number of foil pieces were used for all brazing specimens.

### 2.2. Characterization of Filler Metals

The melting behavior of the fillers was characterized using an STA 449 F3 Jupiter simultaneous thermal analyzer (NETZSCH, Selb, Germany). The filler samples were placed in alumina crucibles and heated from room temperature to 1400 °C at a heating rate of 10 °C min^−1^ under a high-purity argon atmosphere. Static wetting experiments were conducted to evaluate the effect of filler composition on the wettability of the SiC substrates. A small piece of filler metal (approximately 0.1 g) was placed on the polished surface of a SiC substrate. The assembly was placed in a CVI System VII vacuum furnace (Centorr Vacuum Industries, Nashua, NH, USA), heated to 1360 °C, and held for 30 min. After cooling, the apparent contact angle was measured on both sides of each solidified droplet, and the average of the two measurements was reported as the contact angle of the corresponding specimen. The cross-sectional microstructures of the wetting specimens were subsequently examined by scanning electron microscopy coupled with energy-dispersive X-ray spectroscopy SEM–EDS using the procedure described in Section 2.4.

### 2.3. Brazing Process

The SiC brazing couples were assembled in a sandwich configuration (SiC/filler/SiC) using a customized graphite fixture to ensure alignment (Figure 1b). The filler foil was aligned with the 5 × 5 mm^2^ joining surface of the upper SiC block. A uniaxial pressure of 0.1 MPa was applied normal to the joining surfaces during brazing. The brazing experiments were performed in the CVI System VII furnace under a vacuum of approximately 2.7 × 10^−5^ Torr. The assemblies were heated to brazing temperatures ranging from 1320 to 1380 °C at a heating rate of 10 °C min^−1^ and held for 10 min to investigate the effects of brazing temperature on the interfacial reactions. After isothermal holding, the brazed joints were furnace-cooled to room temperature to reduce residual thermal stresses.

### 2.4. Microstructural Characterization

For cross-sectional SEM examination, the specimens after wetting tests and the brazed joints were sectioned perpendicular to the interface and mounted. The exposed cross sections were ground using a 3000-grit diamond grinding disc and polished using a 1 μm diamond polishing agent. No chemical etching was performed. The fracture surfaces were examined without mounting, grinding, polishing, or chemical etching to preserve their original morphologies. The cross-sectional microstructures of the wetting specimens and brazed joints were examined using an SU5000 scanning electron microscope (Hitachi, High-Tech Corporation, Tokyo, Japan) equipped with an energy-dispersive X-ray spectroscopy (EDS) system. The polished cross-sectional microstructures were imaged using a backscattered-electron (BSE) detector, whereas the fracture-surface morphologies were imaged using a secondary-electron detector (SE2). Elemental distributions and local chemical compositions were analyzed by EDS. The phase constituents were analyzed using a D8 ADVANCE X-ray diffractometer (Bruker, AXS GmbH, Karlsruhe, Germany) operated in conventional Bragg–Brentano θ–2θ geometry with Cu Kα radiation. The operating voltage and current were 40 kV and 40 mA, respectively. The diffraction patterns were collected over a 2θ range of 20–80° with a step size of 0.02° and a scanning rate of 2° min^−1^. Phase identification was performed using HighScore Plus software (4.0) with the ICDD PDF-4+ database. Because the incident-beam footprint was larger than the width of the brazed seam, the obtained diffraction pattern represents a composite response from the SiC substrates, brazed seam, and adjacent reaction regions rather than a spatially resolved pattern collected exclusively from the interface.

### 2.5. Mechanical Properties Testing

The shear strengths of the brazed SiC joints were measured using an AG-X Plus electronic universal testing machine (Shimadzu Corporation, Kyoto, Japan) equipped with a customized push-shear fixture, with reference to the testing principles specified in GB/T 31541-2015 and GB/T 11363-2008 [21,22]. As illustrated in Figure 1c, the 8 × 8 × 3 mm^3^ lower SiC block was supported by the fixture, while the load was applied to the 5 × 5 × 3 mm^3^ upper SiC block parallel to the joining interface at a constant crosshead speed of 0.5 mm min^−1^. The shear strength was calculated by dividing the maximum failure load by the nominal brazed area of 25 mm^2^. To evaluate the high-temperature mechanical reliability of the joints, push-shear tests were also conducted at 1000 °C using the same specimen geometry and loading configuration. The specimens were heated to 1000 °C using a high-temperature furnace attached to the testing machine, held for 10 min to promote a uniform temperature distribution, and then tested at the same crosshead speed of 0.5 mm min^−1^. At least five replicate specimens were tested under each experimental condition, and the results are reported as mean values with standard deviations.

## 3. Results and Discussion

### 3.1. Thermophysical Properties and Wetting Behavior of Si-Ti-Al Fillers

**Figure 2:**
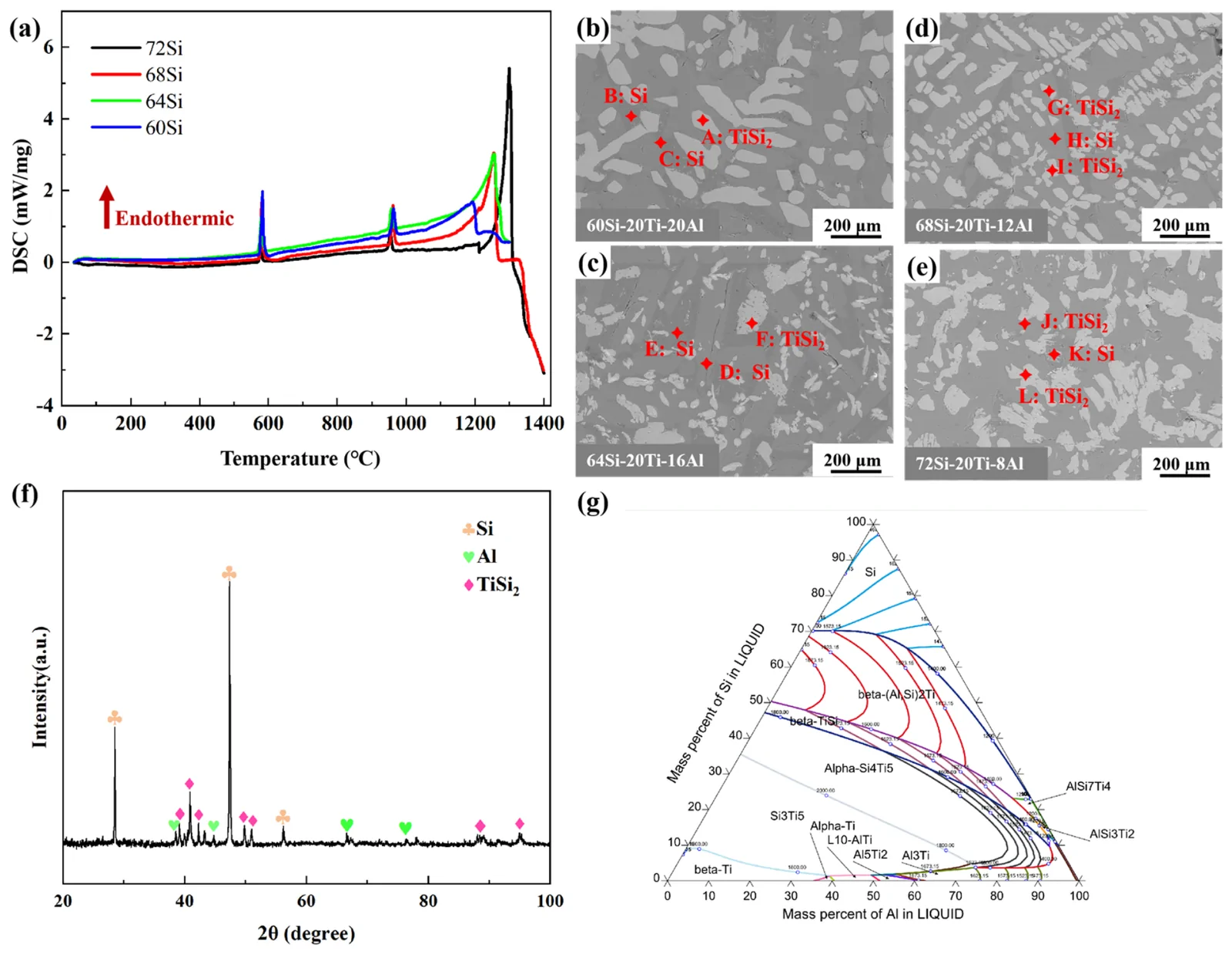
Characterization of the Si–Ti–Al ternary fillers: (**a**) DSC curves of the 72Si, 68Si, 64Si, and 60Si fillers; (**b**–**e**) BSE images of the corresponding filler cross sections; (**f**) XRD pattern of the representative 68Si filler before brazing; and (**g**) liquidus projection of the Si–Ti–Al ternary system expressed in wt.%, showing the nominal compositions of the four fillers.

Four Si-Ti-Al filler compositions were designed along the constant-Ti composition line, with the Ti content fixed at 20 wt.% and Si progressively replaced by Al. Their nominal positions are marked in the calculated Si-Ti-Al ternary liquidus surface projection shown in Figure 2. The investigated compositions are 72Si, 68Si, 64Si, and 60Si, all expressed in wt.%. In this study, with Si as the matrix, an appropriate amount of the active element Ti was introduced to construct a Si-Ti eutectic system, aiming to improve wettability and regulate interfacial reactions [19,20,22,23]. Concurrently, Al was incorporated to tailor the melting point and enhance the plasticity of the alloy, thereby constructing a novel Si-Ti-Al ternary brazing filler system for SiC ceramic joining. Building upon this fundamental design, a series of compositions was strategically optimized based on the Si-Ti-Al ternary phase diagram [24]. With the Ti content fixed at 20 wt.%, progressively substituting Si with Al yields a dual effect on the metallurgical behavior of the fillers. On one hand, the increased Al content gradually lowers the melting point and reduces the liquid viscosity, which significantly enhances the fluidity and gap-filling capacity of the molten filler. On the other hand, the reduction in Si content at a constant Ti level effectively increases the Ti/Si atomic ratio, which thermodynamically elevates the driving force for the formation of Ti-Si intermetallic compounds (e.g., TiSi_2_) within the liquid phase. Additionally, the elevated Al content promotes the formation of Al-Ti intermetallic phases [25]. Consequently, an excessively low Si content would result in an overabundance of coarse TiSi_2_ precipitates, severely embrittling the joint. Therefore, an optimal composition window must be identified to balance the beneficial effect of improved fluidity against the detrimental effect of excessive intermetallic precipitation. To compare the melting behavior of the fillers and determine an appropriate brazing-temperature range, the four filler compositions were analyzed by DSC. As shown in Figure 2a, the liquidus temperatures of the 72Si, 68Si, 64Si, and 60Si fillers were determined to be 1312, 1272, 1266, and 1210 °C, respectively. The liquidus temperature generally decreased as Si was progressively replaced by Al, with the most pronounced decrease observed for the 60Si filler. Because the liquidus temperatures of all four fillers were below the investigated brazing-temperature range of 1320–1380 °C, the fillers could become fully molten under the selected joining conditions. The broad endothermic peak at ~1250–1300 °C represents the main melting of the filler metal. Consistent with the phase diagram design, as Al content increases to substitute for Si, the main melting peak progressively shifts to lower temperatures, indicating a gradual decrease in the liquidus temperature of the fillers. Although this trend somewhat compromises the high temperature structural stability of the solidified fillers, it significantly enhances the fluidity and gap-filling capability of the molten brazing alloy. Given that robust interfacial reaction between the filler and SiC substrate requires elevated temperatures to overcome the kinetic barrier, a brazing temperature range of 1320–1380 °C was selected to ensure adequate metallurgical activity at the joint interface. The as-prepared filler foils were examined by BSE imaging and site-specific EDS analysis before the wetting and brazing experiments, as shown in Figure 2b–e and Table 2. The analysis positions A–C, D–F, G–I, and J–L correspond to the 60Si, 64Si, 68Si, and 72Si fillers, respectively. The fillers exhibit compositionally distinct Si-rich and Ti–Si-rich regions, while the local Al contents vary among the analyzed regions and filler compositions. These results demonstrate that the multiphase microstructures and elemental partitioning were already present in the as-prepared fillers following arc melting and solidification, rather than being generated exclusively during the subsequent wetting or brazing treatments. The relative amounts and morphologies of these constituent regions vary with the nominal filler composition. To identify the crystalline phases present before brazing, the representative 68Si filler was further examined by XRD. As shown in Figure 2f, Si, TiSi_2_, and residual Al were identified in this representative filler, consistent with the SEM-EDS observations. The combined results indicate that the as-prepared 68Si filler contains Si, TiSi_2_, and residual Al before brazing.

**Figure 3:**
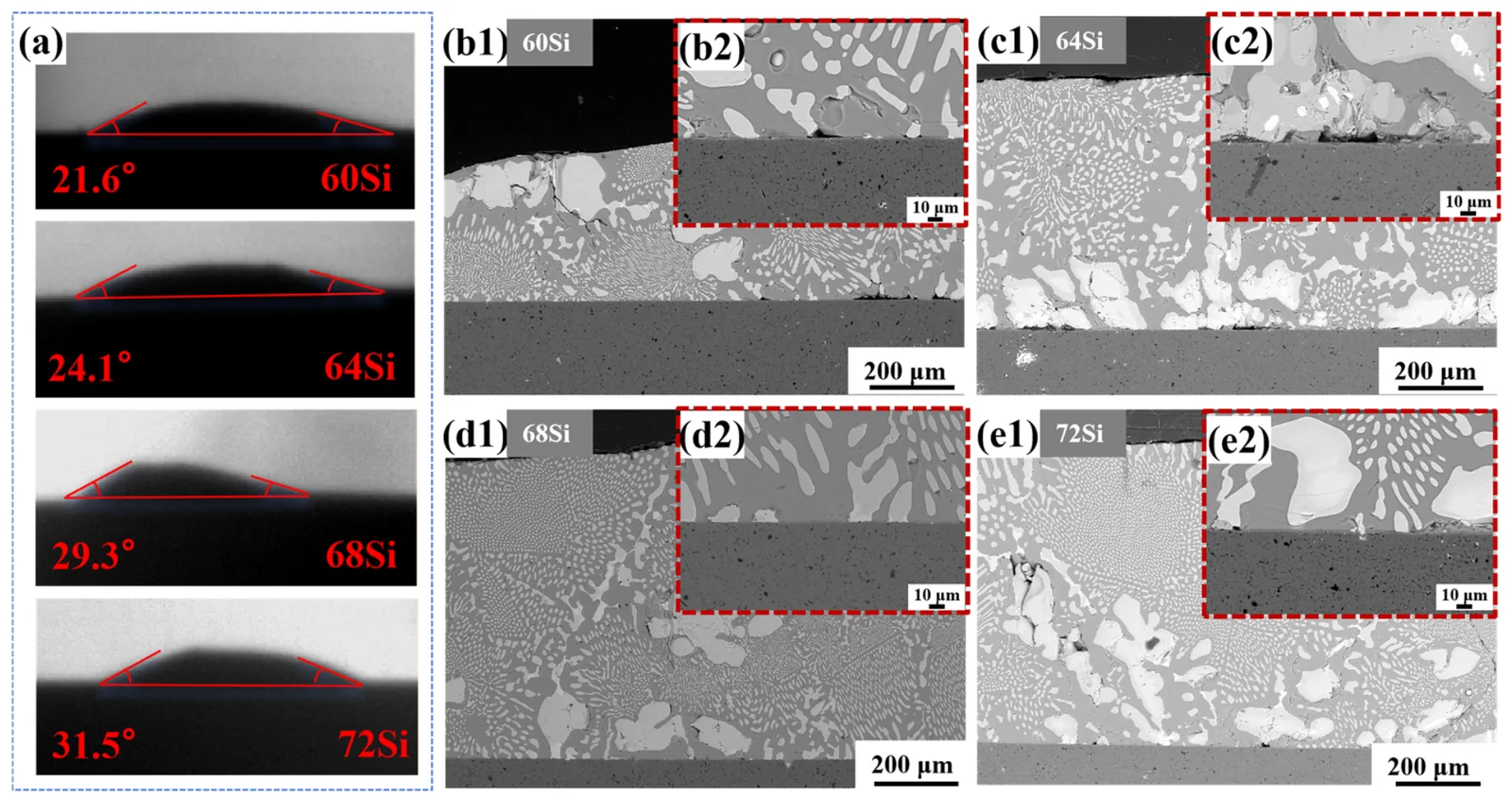
Wetting behavior of the Si–Ti–Al fillers on the SiC substrates: (**a**) apparent contact angles; (**b1**,**c1**,**d1**,**e1**) cross-sectional BSE images of the wetting specimens produced using the 60Si, 64Si, 68Si, and 72Si fillers, respectively; and (**b2**,**c2**,**d2**,**e2**) corresponding high-magnification BSE images of the filler/SiC interfacial regions.

Macroscopic observations indicate that all four fillers exhibit favorable wetting behavior on the SiC surface (as shown in Figure 3a). Specifically, the equilibrium contact angles for the 72Si, 68Si, 64Si, and 60Si fillers are 31.5°, 29.3°, 24.1°, and 21.6°, respectively, indicating progressively improved wettability with decreasing Si content. This trend is attributed to the combined effect of reduced Si content and increased Al content, which lowers the melting point and liquid viscosity of the filler, thereby enhancing the fluidity and spreading of the molten alloy. Previous studies have similarly demonstrated favorable wetting and spreading of Si–Ti-based melts on SiC and SiC-based composites (Figure 3b1–e2), confirming the suitability of Si-rich Ti-containing fillers for joining SiC materials [20,22].

To further elucidate the wetting characteristics, the interfacial microstructures were examined in detail, as shown in Figure 3a–d2, with high-magnification BSE images of representative filler/SiC interfacial regions included as insets. The high-magnification images show that the interfaces formed by the 72Si and 68Si fillers are relatively continuous at the examined scale, whereas localized gaps and interfacial discontinuities are observed for the 60Si and 64Si fillers, particularly for the 64Si filler. As the Si content is further reduced, pronounced changes are also observed in the morphology and spatial distribution of the bright phases. In the 64Si filler (Figure 3b1,b2), numerous coarse bright blocky features are present near the SiC interface, accompanied by localized discontinuities at the contact boundary. In contrast, the excessively large bright blocky features in the 60Si filler (Figure 3a) are located predominantly in the upper portion of the solidified droplet rather than being concentrated directly at the filler/SiC interface. These features exhibit BSE contrast and morphology similar to those of the Ti–Si-rich phases identified in the representative 68Si filler; however, their exact phase identities in the 64Si and 60Si fillers cannot be conclusively established without corresponding local EDS or XRD evidence. Because titanium silicides are intrinsically brittle, coarse Ti–Si-rich phases, if present, may act as local stress concentrators during cooling and could facilitate crack initiation in the surrounding matrix [26]. However, such microcracks are not clearly resolved at the magnification used in Figure 3a–b2. Thus, although lowering the Si content improves macroscopic wettability, the accompanying changes in the size and spatial distribution of the bright blocky phases may adversely affect the microstructural integrity of the solidified filler, necessitating a balanced composition design.

### 3.2. Microstructure of SiC Joints Brazed with 68Si Filler

**Figure 4:**
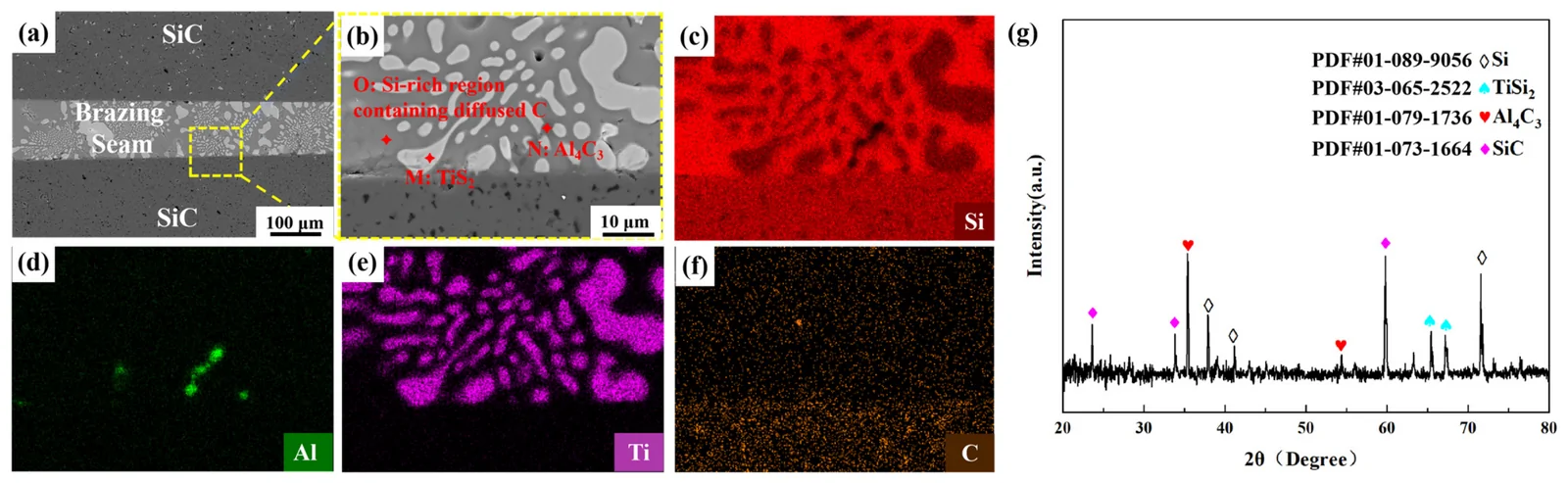
Typical interfacial microstructure and elemental distributions of the SiC joint brazed with the 68Si filler at 1360 °C for 10 min: (**a**,**b**) BSE images of the polished joint cross section; (**c**–**f**) EDS elemental maps; and (**g**) XRD pattern of the irradiated joint region.

Based on a comprehensive consideration of the melting characteristics, wettability, and solidified microstructure of the four fillers, the 68Si filler was selected as the representative composition for the subsequent brazing-temperature study. TiSi_2_-containing phases were observed in all four fillers and were therefore not used as the sole criterion for composition selection. Although the 60Si and 64Si fillers exhibited smaller contact angles, relatively pronounced coarse-phase aggregation was observed in their solidified droplets, together with local interfacial discontinuities in the 64Si filler. In contrast, the 72Si filler exhibited a higher liquidus temperature of 1312 °C and a larger contact angle of 31.5°. The 68Si filler exhibited adequate wettability, with a contact angle of 29.3°, and its liquidus temperature of 1272 °C provided a suitable processing window for the subsequent brazing experiments conducted at 1320–1380 °C. Moreover, its constituent phases were comparatively uniformly distributed within the solidified droplet. Therefore, the 68Si filler provided an appropriate overall balance among melting characteristics, wettability, and microstructural uniformity. Figure 4 presents the typical microstructure and EDS elemental mapping of the SiC/SiC joint brazed with the 68Si filler at 1360 °C for 10 min. Examination of the joint structure in Figure 4a,b shows that the joint is continuous without obvious defects such as porosity or microcracks, indicating that the brazing filler has good fluidity to fully wet the SiC. Previous studies have shown that Ti-containing fillers can react with SiC during active brazing and form TiC-containing reaction products at the interface [27]. As shown in the elemental distribution maps in Figure 4c–f, the interface between the brazed seam and the SiC substrate is continuous and relatively straight. No continuous Ti-rich/C-rich reaction layer was detected at the interface within the spatial resolution of SEM–EDS, suggesting that the formation of a continuous TiC reaction layer was limited under the investigated brazing conditions. The Al elemental map in Figure 4d reveals localized Al enrichment near the filler/SiC interface. The EDS result for point N shows an Al–C-rich composition with an Al/C atomic ratio of approximately 1.46, which is close to the stoichiometric ratio of Al_4_C_3_. Combined with the Al_4_C_3_ reflections identified in the XRD pattern in Figure 4g, these results indicate the presence of discrete Al_4_C_3_-containing regions within the brazed joint. The discontinuous distribution of these regions suggests that Al_4_C_3_ is a minor reaction product rather than a continuous interfacial reaction layer.

**Figure 5:**
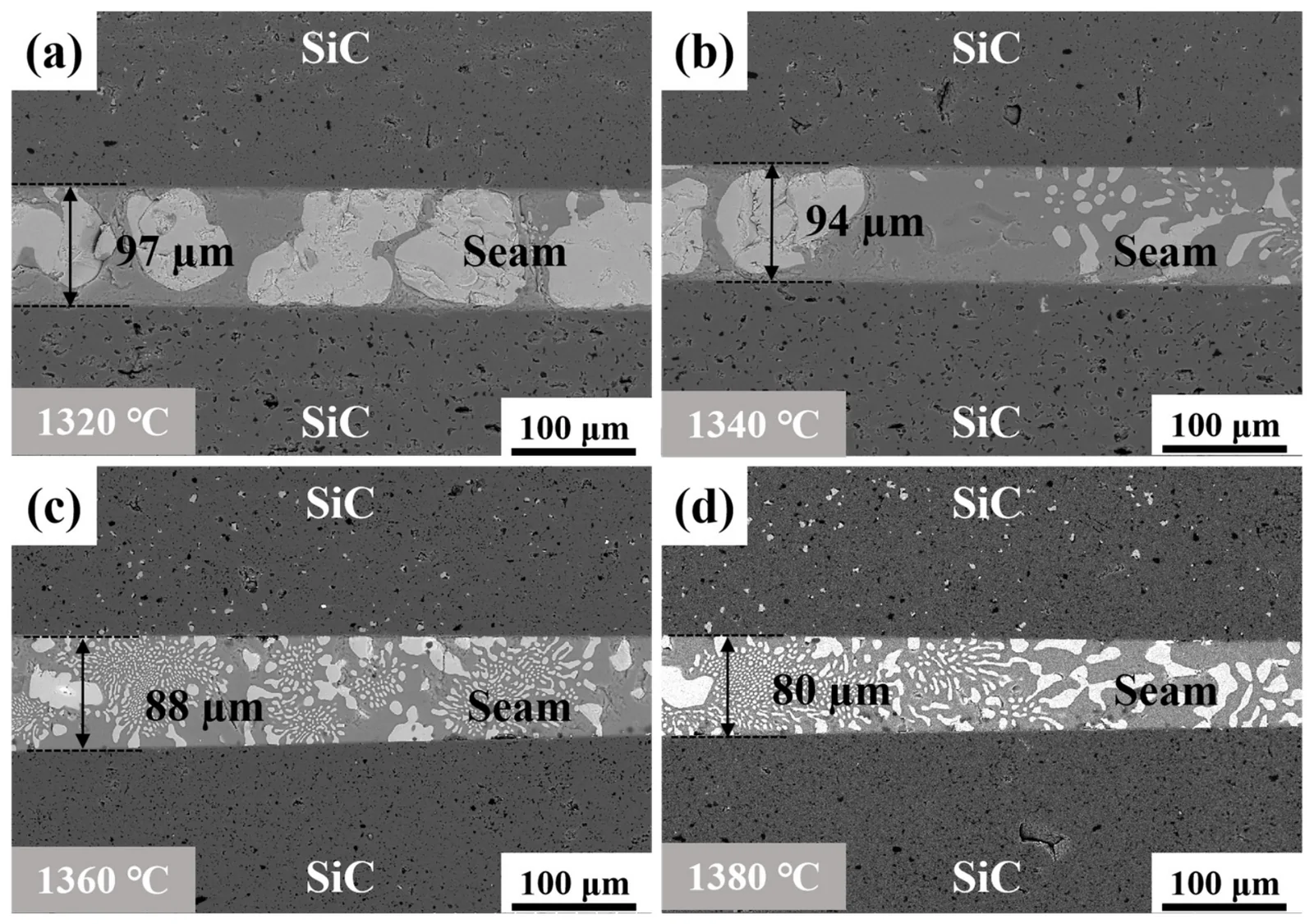
BSE images showing the cross-sectional microstructural evolution of the SiC joints brazed with the 68Si filler at different temperatures: (**a**) 1320 °C, (**b**) 1340 °C, (**c**) 1360 °C, and (**d**) 1380 °C.

To investigate the effect of process parameters on the joint microstructure, the brazing temperature was varied and the corresponding microstructural evolution was analyzed, as shown in Figure 5. It is evident that the brazing temperature profoundly influences the morphology, size, and spatial distribution of the precipitated phases within the brazed seam. As the brazing temperature increases, the brazed seam gradually becomes thinner, with representative thicknesses of approximately 97, 94, 88, and 80 μm at 1320, 1340, 1360, and 1380 °C, respectively. This evolution is associated with the enhanced fluidity and spreading of the molten filler at elevated temperatures, which facilitates its lateral flow under the applied joining pressure. At relatively lower brazing temperatures of 1320 °C and 1340 °C (Figure 5a,b), the microstructures are dominated by excessively coarse and highly agglomerated bright-white blocky phases (primary TiSi_2_). The insufficient superheat restricts atomic mobility and liquid fluidity, leading to localized elemental accumulation and the severe coarsening of these primary intermetallic compounds.

When the brazing temperature increases to 1360 °C (Figure 5c), a remarkable microstructural refinement is observed. The massive blocky phases are significantly reduced in size and become uniformly dispersed. Concurrently, a large volume fraction of highly regular, fine coral-like eutectic clusters precipitates extensively in the central region of the seam, the EDS results are shown in Table 3.

As the brazing temperature is further elevated to 1380 °C (Figure 5d), a higher volume fraction of fine coral-like eutectic clusters precipitates in the central region of the brazed seam, whereas blocky compounds form in the microstructure adjacent to the SiC substrate. Because SiC is a highly covalent ceramic with a dense, rigid structure, the diffusion of foreign atoms into its lattice is extremely limited. Ti atoms are confined near the solid–liquid boundary. Consequently, during the cooling stage, the primary blocky brittle phase (TiSi_2_) preferentially re-nucleates and severely coarsens along the SiC interface [28].

**Figure 6:**
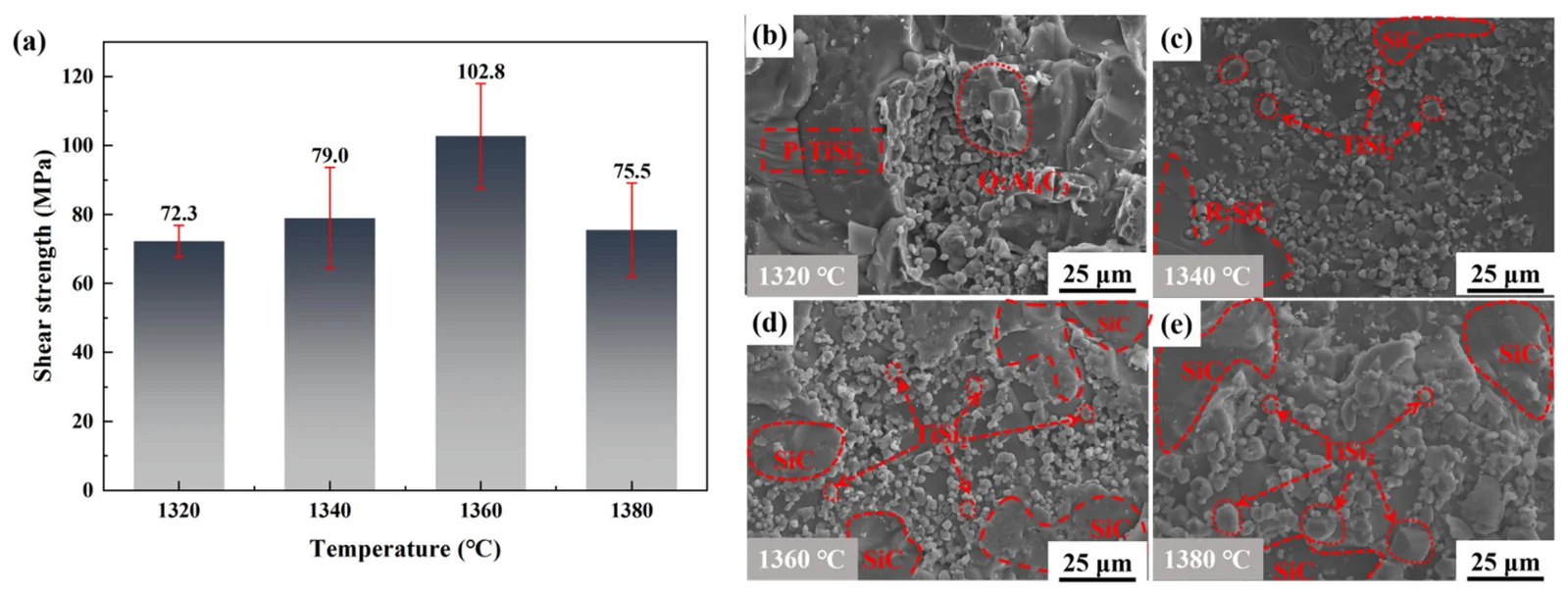
Room-temperature shear properties and corresponding fracture morphologies of the SiC joints brazed with the 68Si filler at different temperatures: (**a**) room-temperature shear strength; (**b**–**e**) SE2 images of the fracture surfaces of the joints brazed at (**b**) 1320 °C, (**c**) 1340 °C, (**d**) 360 °C, and (**e**) 1380 °C.

To evaluate the mechanical properties of the joints, the room temperature shear strength of the joints brazed at different temperatures was tested, and the results are shown in Figure 6a. The shear strength exhibits a trend, initially increasing and then decreasing as the brazing temperature rises. At the relatively low brazing temperature of 1320 °C, the joint exhibits a lower shear strength of 72.3 MPa. This is primarily attributed to the insufficient thermal input, which restricts atomic diffusion and dynamic supercooling, thereby leading to the excessive coarsening and severe agglomeration of the TiSi_2_ phases. Under shear loading, these massive, contiguous brittle phases act as hazardous stress concentrators and preferential sites for crack initiation, ultimately leading to the premature failure of the joint at a lower stress level. As the temperature increases to 1340 °C, the enhancement in liquid fluidity effectively optimizes the solidified microstructure of the brazed seam, thereby yielding an increased shear strength of 79.0 MPa. When the brazing temperature reaches 1360 °C, the shear strength peaks at an impressive value of 102.8 MPa. The high load-bearing capacity of this joint is associated with the combined effects of its favorable seam geometry, refined multiphase microstructure, and continuous interface. The extensive precipitation of fine, highly regular coral-like eutectic clusters, combined with a defect-free and smooth interface, provides an excellent stress-accommodating capacity. This optimized multiphase configuration effectively absorbs fracture energy and deflects crack propagation, thereby maximizing the macroscopic strength of the joint. A similar dependence of joint strength on the morphology, size, and distribution of the reaction products has been reported for SiC joints brazed with Si–Ti-based fillers, in which microstructural refinement and suppression of coarse brittle phases were beneficial to the mechanical properties [15,28]. However, when the brazing temperature is further increased to 1380 °C, the brazed-seam thickness decreases to approximately 80 μm, while the shear strength decreases to 75.5 MPa. At this temperature, the primary blocky TiSi_2_ phases re-nucleate and undergo pronounced coarsening along the SiC interface. The resulting heterogeneous microstructure promotes localized stress concentration and premature interfacial fracture, leading to the reduction in shear strength.

To further elucidate the failure mechanisms and corroborate the mechanical property evolution, the fracture surfaces of the brazed joints after the room temperature shear tests were characterized, as shown in Figure 6b–e. Representative fracture-surface features marked as points P–R in Figure 6b,c were further analyzed by EDS, and the corresponding elemental compositions and possible phase assignments are summarized in Table 4. Points P and Q exhibit pronounced Ti–Si and Al–C enrichment, respectively, supporting the assignment of these features to TiSi_2_ and Al_4_C_3_ in conjunction with the XRD results shown in Figure 4g. Point R exhibits pronounced enrichment of Si and C, supporting its assignment as a locally exposed SiC-substrate region. The spatially separated SiC-containing regions visible in Figure 6c–e do not represent isolated SiC particles dispersed within the brazed seam, nor do they indicate penetration of the molten filler into pores of the SiC substrate. Instead, the three-dimensional crack path locally alternates among the brazed seam, the filler/SiC interface, and the adjacent SiC substrate. Consequently, discontinuously exposed portions of the continuous SiC substrate appear as spatially separated regions in the two-dimensional fracture-surface images. The variations in fracture morphology intuitively reflect the profound impact of the brazing temperature on the crack propagation path and interfacial bonding state. For the joint brazed at a relatively low temperature of 1320 °C (Figure 6b), the fracture surface is primarily characterized by massive, exposed TiSi_2_ blocks and a minor amount of agglomerated Al_4_C_3_ particles on relatively flat cleavage planes. Because the primary intermetallic compounds fail to be adequately refined, cracks rapidly initiate at these massive brittle blocks and propagate unhindered through the brazed seam, exhibiting a typical brittle fracture mode. This crack propagation path consumes minimal fracture energy, thereby accounting for its low shear strength. As the brazing temperature increases to 1340 °C (Figure 6c), the fracture surface exhibits partially exposed SiC regions and relatively refined Al_4_C_3_ particles. However, the overall morphology remains noticeably flat. The lack of effective crack deflection or significant plastic deformation indicates that the failure is still dominated by low-energy cleavage fracture along the interface and brazed seam. As the brazing temperature increases to 1360 °C (Figure 6d), a remarkable microstructural refinement is observed. The TiSi_2_ and the Al_4_C_3_ particles on the fracture surface become significantly finer and more uniformly distributed. This microstructural feature indicates that the highly refined and homogeneous multiphase configuration effectively deflects crack propagation, thereby consuming a substantial amount of fracture energy. More importantly, obvious areas of SiC substrate tearing are distinctly visible. This indicates that the interfacial metallurgical bonding—strengthened by the trace Al_4_C_3_ anchoring—is exceptionally robust, leading to a high-energy mixed fracture mode. However, when the brazing temperature is further elevated to 1380 °C (Figure 6e), the fracture surface morphology deteriorates significantly. It exhibits broad and flat cleavage facets accompanied by severely coarsened and agglomerated TiSi_2_ blocks as well as enlarged Al_4_C_3_ particles. The extensive exposure of the SiC substrate suggests that the crack preferentially propagates along the embrittled SiC/filler interface, reflecting a low-energy brittle fracture mode caused by excessive interfacial degradation.

**Figure 7:**
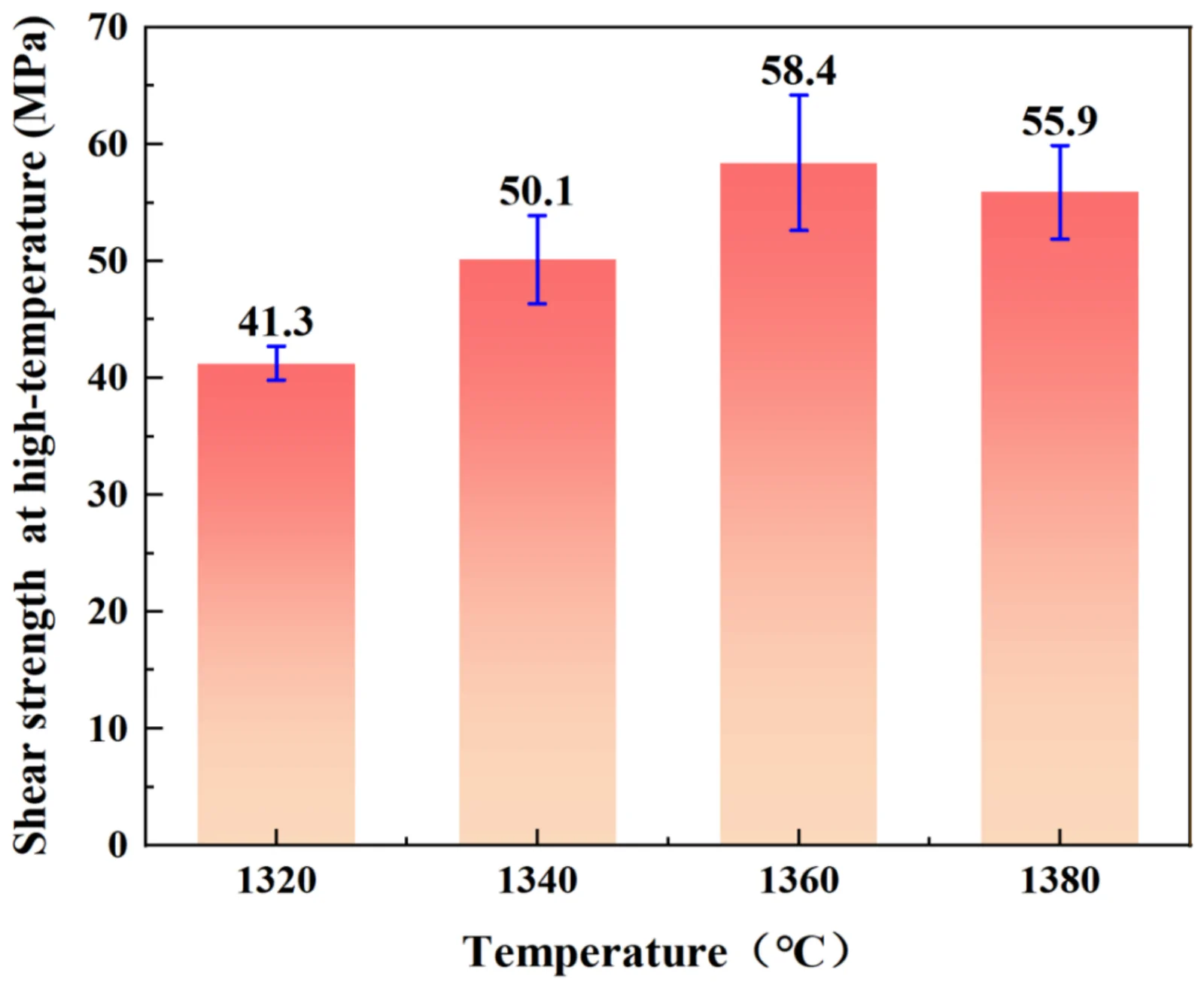
High temperature shear strength of the joints tested at 1000 °C.

**Figure 8:**
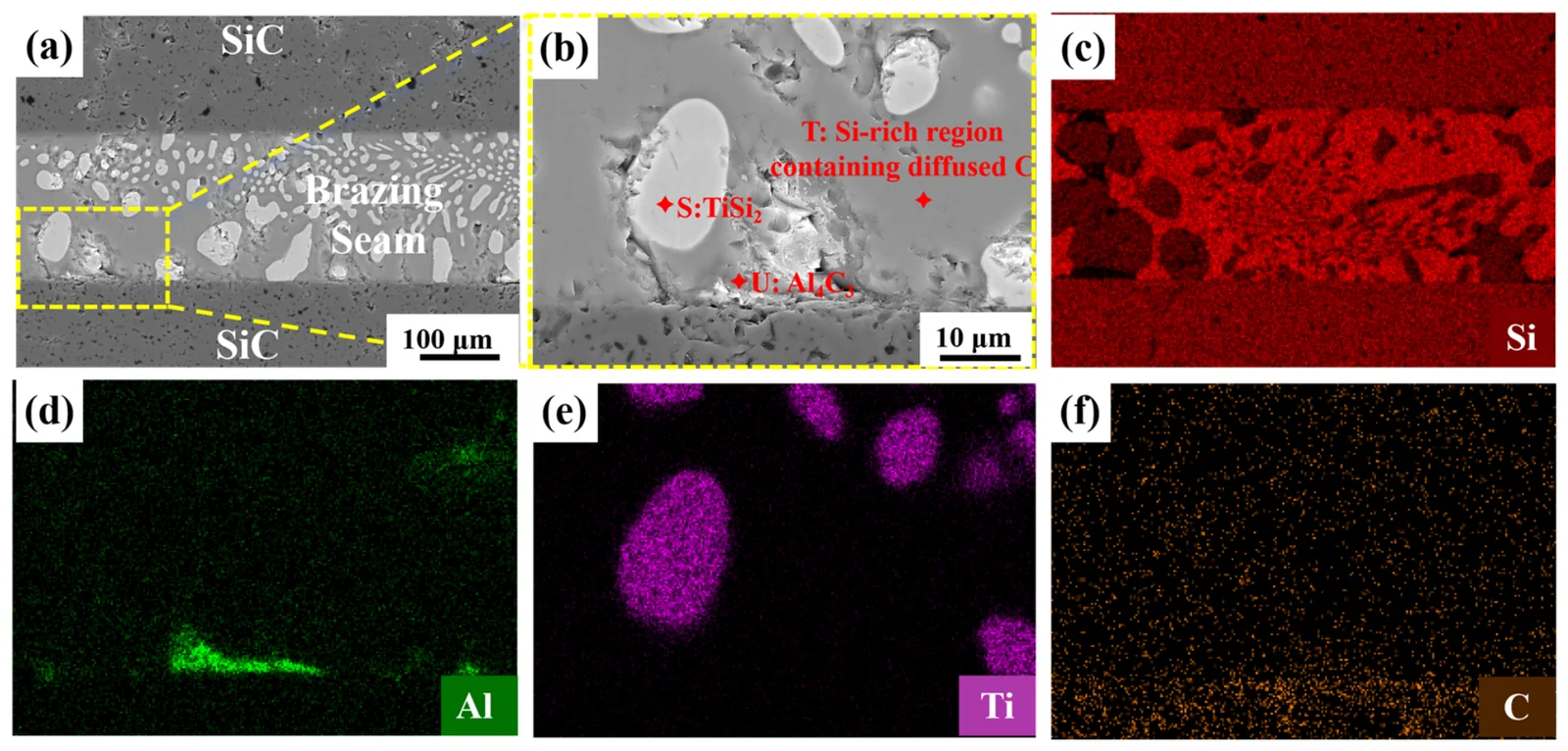
Typical interfacial microstructure and elemental distributions of the SiC joint brazed with the 68Si filler at 1360 °C after isothermal holding at 1000 °C for 10 min: (**a**,**b**) BSE images of the polished joint cross section; (**c**–**f**) EDS maps acquired from the region shown in (**b**).

To further evaluate the service reliability of the brazed joints in extreme environments, the high temperature shear strengths of the joints brazed at 1320–1380 °C were tested at 1000 °C. As shown in Figure 7, the high temperature shear strength exhibits the same trend as the room temperature results. With the increase in brazing temperature, the joint strength initially increases and then decreases, reaching a maximum value of 58.4 MPa at 1360 °C. To elucidate the underlying mechanism of this robust high temperature performance, the microstructural evolution of the optimal joint subjected to an isothermal heat treatment at 1000 °C for 10 min was systematically investigated, as shown in Figure 8.

Comparison with the corresponding room-temperature results shows that the shear strengths measured at 1000 °C are reduced. This reduction is associated with both the direct effect of the elevated testing temperature on the load-bearing response of the joint and the microstructural evolution occurring during thermal exposure. As shown in Figure 8a,b, some of the primary blocky brittle phases in the brazed seam undergo partial re-agglomeration and coarsening, whereas a considerable proportion of the fine, regular coral-like eutectic clusters remains preserved. The elevated testing temperature and the observed microstructural evolution may both contribute to the reduction in shear strength.

Furthermore, combined with the EDS elemental mapping results (Figure 8c–f), the Al map (Figure 8d) reveals that the interfacial Al_4_C_3_ precipitates exhibit noticeable coarsening after the 1000 °C thermal exposure. This growth of Al_4_C_3_ indicates a thermally activated continuation of the interfacial reaction between the residual Al in the filler and the SiC substrate. The coarsened Al_4_C_3_ phases act as localized stress concentrators, which also contribute to the reduction in high temperature strength. Nevertheless, the absence of a continuous TiC layer ensures that the overall interfacial integrity remains preserved. The EDS results in Table 5 show that the Si-rich region at point T contains a measurable amount of C (19.12 at.%). Together with the C distribution in Figure 8f, this suggests continued diffusion of C from the SiC substrate into the brazed seam during thermal exposure at 1000 °C, resulting in local C enrichment within the Si-rich matrix.

**Figure 9:**
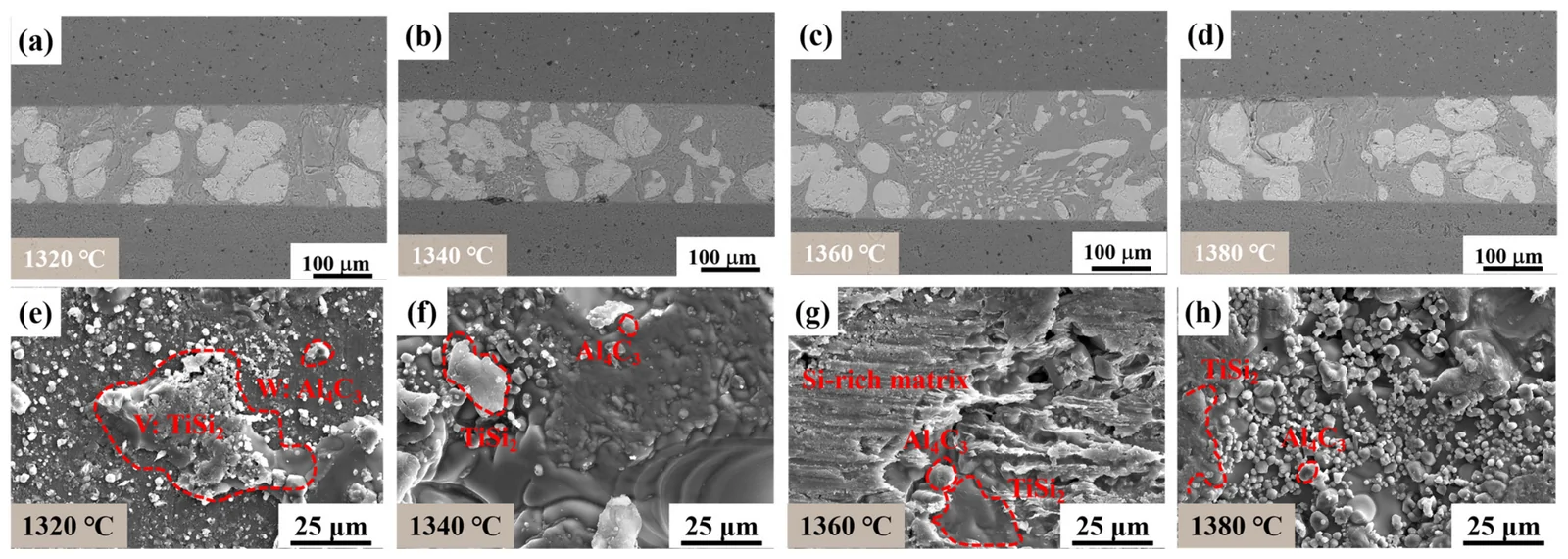
Cross-sectional microstructures and corresponding fracture-surface morphologies of the SiC joints brazed with the 68Si filler after testing at 1000 °C: (**a**–**d**) BSE images of the polished cross sections of the joints brazed at (**a**) 1320 °C, (**b**) 1340 °C, (**c**) 1360 °C, and (**d**) 1380 °C; (**e**–**h**) SE2 images of representative alloy-dominated fracture-surface regions of the corresponding joints.

Furthermore, as illustrated in Figure 9, the microstructural evolution of the joints brazed at other temperatures following the 1000 °C thermal exposure provides profound insights into their varied high temperature strengths. For the joints prepared at relatively lower temperatures (1320 °C and 1340 °C, Figure 9a,b), the brittle compounds within the central region of the brazed seam also experience severe coarsening and agglomeration. This is primarily because the insufficient thermal input during the initial brazing stage prevents the adequate refinement of the primary TiSi_2_ phases, leaving massive brittle particles in the center of the seam. During the subsequent isothermal holding, the pre-existing coarse particles provide preferential sites for further growth. This coarsening behavior is consistent with the Ostwald-ripening mechanism, in which larger particles grow at the expense of smaller particles to reduce the total interfacial energy [29,30]. Consequently, the surrounding fine eutectic structures are gradually consumed, resulting in pronounced microstructural coarsening. This large-scale embrittlement in the central region severely degrades the load-bearing capacity of the matrix, directly accounting for their lowest high temperature shear strengths (41.3 and 50.1 MPa). In contrast, the coarsening and agglomeration in the 1380 °C joint (Figure 9d) exhibit a distinct segregation characteristic adjacent to the interface. Because the initially excessive heat input confines a large volume of primary blocky phases near the SiC solid–liquid boundary, the same Ostwald ripening and coalescence mechanisms drive severe microstructural degradation and macro-agglomeration at the interface, reducing its strength to 55.9 MPa.

To further elucidate the high-temperature failure mechanisms, representative alloy-dominated regions of the fracture surfaces after the shear tests at 1000 °C were characterized, as shown in Figure 9e–h. Representative features marked as points V and W in Figure 9e were further analyzed by site-specific EDS, and the corresponding elemental compositions are summarized in Table 6. Point V exhibits pronounced enrichment of Ti and Si, with an Si/Ti atomic ratio close to 2.0. Combined with the TiSi_2_ reflections detected by XRD, this feature is identified as TiSi_2_. Point W is enriched in Al and C and is identified as Al_4_C_3_ in conjunction with the corresponding XRD reflections. Although separate EDS analyses were not conducted on every feature visible in Figure 9f–h, the phase assignments were made by combining the representative fracture-surface EDS results, XRD phase identification, and the corresponding morphological characteristics.

For the joint brazed at 1320 °C (Figure 9e), the fracture surface is rough and heterogeneous, with coarse TiSi_2_ blocks and locally agglomerated Al_4_C_3_ distributed within the brazed seam. These coarse brittle phases provide preferential sites for crack initiation and propagation, consistent with the relatively low high-temperature shear strength of this joint. At 1340 °C (Figure 9f), relatively broad matrix regions are observed together with locally retained TiSi_2_ and dispersed Al_4_C_3_. The reduced extent of coarse-phase agglomeration corresponds to the moderate improvement in shear strength.

For the joint brazed at 1360 °C (Figure 9g), the selected fracture region is dominated by the Si-rich matrix of the brazed seam, together with comparatively fine TiSi_2_ and localized Al_4_C_3_. The presence of these characteristic brazed-seam constituents indicates that the selected fracture area is predominantly located within the brazed seam. The pronounced tearing ridges and irregular crack-propagation path indicate increased crack deflection and fracture-energy consumption, consistent with the highest shear strength retained by this joint at 1000 °C. When the brazing temperature is increased to 1380 °C (Figure 9h), TiSi_2_ and Al_4_C_3_ exhibit pronounced coarsening and local aggregation. The resulting heterogeneous distribution of the brittle phases promotes local stress concentration and facilitates crack propagation through the brazed seam, thereby reducing the high-temperature shear strength.

## 4. Conclusions

In this paper, a novel Si-Ti-Al ternary alloy was used to realize the highly reliable brazing connection of SiC ceramics, and the microstructural evolution, phase competition, and failure mechanisms of the joints at room and elevated temperatures were investigated.

(1) Increasing the Si content promoted the incorporation of Ti into Ti–Si phases within the filler and brazed seam. This compositional design reduced the tendency for excessive Ti–SiC interfacial reactions while maintaining favorable melting behavior and wettability.
(2) No continuous TiC layer or graphite was detected at the interface within the spatial resolution of the employed characterization methods. The joint was primarily bonded through a Si-rich matrix containing dispersed Ti–Si phases, together with a minor amount of interfacial Al_4_C_3_.
(3) The 68Si filler has a liquidus temperature of 1272 °C. At 1360 °C, the joint consists of a Si matrix, TiSi_2_ dispersoids, residual Al, and minor interfacial Al_4_C_3_; no continuous TiC layer or graphite was detected. The extensive precipitation of coral-like eutectic clusters yields a peak room-temperature shear strength of 102.8 MPa.
(4) The joint exhibits excellent high temperature reliability, maintaining a shear strength of 58.4 MPa at 1000 °C. During high-temperature exposure, despite the Ostwald ripening and partial agglomeration of primary blocky phases and the coarsening of interfacial Al_4_C_3_ precipitates, the robust retention of the fine eutectic clusters ensures the excellent high temperature structural stability of the joint.

## Figures and Tables

**Table 1:** Designations and nominal chemical compositions of the Si–Ti–Al filler alloys (wt.%).

Filler	Si	Ti	Al
72Si	72	20	8
68Si	68	20	12
64Si	64	20	16
60Si	60	20	20

**Table 2:** EDS elemental compositions of the regions marked in Figure 2 (at.%).

Point	Al	Ti	Si	Possible Phase
A	0.06	35.44	64.50	TiSi_2_
B	0.00	0.11	99.89	Si
C	0.00	0.26	99.74	Si
D	0.45	0.00	99.55	Si
E	0.30	0.00	99.70	Si
F	9.57	33.16	57.27	TiSi_2_
G	6.52	31.76	61.72	TiSi_2_
H	0.00	0.15	99.85	Si
I	8.36	34.17	57.47	TiSi_2_
J	10.68	33.07	56.25	TiSi_2_
K	0.00	0.15	99.85	Si
L	6.52	31.76	61.72	TiSi_2_

**Table 3:** EDS elemental compositions of the regions marked in Figure 4 (at.%).

Point	Al	Ti	Si	C	Possible Phase
M	0.00	29.36	56.87	13.77	TiSi_2_
N	52.76	0.58	10.62	36.04	Al_4_C_3_
O	0.00	0.21	76.31	23.47	Si-rich region containing diffused C

**Table 4:** EDS elemental compositions of the regions marked in Figure 6 (at.%).

Point	Al	Ti	Si	C	Possible Phase
P	2.53	27.47	48.42	21.59	TiSi_2_
Q	47.83	0.60	4.05	47.51	Al_4_C_3_
R	0.00	0.21	67.41	32.38	SiC

**Table 5:** EDS elemental compositions of the regions marked in Figure 8 (at.%).

Point	Al	Ti	Si	C	Possible Phase
S	0.23	30.20	56.10	13.47	TiSi_2_
T	0.00	4.00	76.88	19.12	Si-rich region containing diffused C
U	31.14	0.19	30.05	38.63	Al_4_C_3_

**Table 6:** EDS elemental compositions of the regions marked in Figure 9 (at.%).

Point	Al	Ti	Si	C	Possible Phase
V	7.35	25.83	47.75	19.08	TiSi_2_
W	16.13	0.08	49.07	34.72	Al_4_C_3_

## Data Availability

The original contributions presented in this study are included in the article. Further inquiries can be directed to the corresponding author.
